# Potential New Approaches for Diagnosis of Alzheimer's Disease and Related Dementias

**DOI:** 10.3389/fneur.2020.00496

**Published:** 2020-06-05

**Authors:** R. Scott Turner, Terry Stubbs, Don A. Davies, Benedict C. Albensi

**Affiliations:** ^1^Department of Neurology, Georgetown University, Washington, DC, United States; ^2^ActivMed, Practices & Research, Methuen, MA, United States; ^3^Division of Neurodegenerative Disorders, St Boniface Hospital Research, University of Manitoba, Winnipeg, MB, Canada; ^4^Department of Pharmacology & Therapeutics, Max Rady College of Medicine, University of Manitoba, Winnipeg, MB, Canada

**Keywords:** memory, dementia, diagnostic, novel, clinic, Alzheimer

## Abstract

Dementia is an umbrella term—caused by a large number of specific diagnoses, including several neurodegenerative disorders. Alzheimer's disease (AD) is now the most common cause of dementia in advanced countries, while dementia due to neurosyphilis was the leading cause a century ago. Many challenges remain for diagnosing dementia definitively. Some of these include variability of early symptoms and overlap with similar disorders, as well as the possibility of combined, or mixed, etiologies in some cases. Newer technologies, including the incorporation of PET neuroimaging and other biomarkers (genomics and proteomics), are being incorporated into revised diagnostic criteria. However, the application of novel diagnostic methods at clinical sites is plagued by many caveats including availability and access. This review surveys new diagnostic methods as well as remaining challenges—for clinical care and clinical research.

## Introduction

Alzheimer's disease (AD), the most common cause of dementia, is now the 6th leading cause of death in the United States (Centers for Disease Control and Prevention). There are close to 50 million individuals with AD globally, and ~ 6 million individuals in the United States alone (Alzheimer's Disease International). Dementia is an umbrella term and may be caused by many disorders, including several neurodegenerative diseases. The differential diagnosis of dementia in older individuals typically includes AD, vascular dementia (VaD), dementia with Lewy bodies (DLB), Parkinson's disease dementia (PDD), frontotemporal dementia (FTD), and mixed dementias. Infectious etiologies of cognitive decline include neurosyphilis and HIV, and must be ruled out in the work-up of selected individuals. By definition, dementia requires a decline in more than one cognitive domain—memory, praxis, gnosis, language, visuospatial skills, executive function—and may also be accompanied by one or more behavioral disorders—depression, anxiety, personality changes, hallucinations, and delusions. The word dementia, taken from Latin, means “to take away one's mind,” and is accompanied by significant social stigma in all cultures. The Diagnostic and Statistical Manual of Mental Disorders 5 (DSM-5, 2013) suggested deleting this demeaning term from our medical vocabulary, and proposed instead *major neurocognitive disorder* in order to minimize stigma and discrimination toward those affected (similar to the discontinuation of the demeaning term *mongoloid* in favor of Down syndrome). However, this attempt has mostly failed and most practitioners and advocacy groups still use the term dementia when referring to Alzheimer's disease and related conditions.

Although the Greeks and Romans were well aware of dementia and associated it with aging, Alzheimer's disease (AD) was first reported in 1906 by the psychiatrist Alois Alzheimer. While at a Frankfurt hospital in Germany, Dr. Alzheimer examined his patient Auguste Deter, a 51 year-old woman, and described her as having progressive sleep and memory disturbances, confusion, paranoia, and aggression. Five years later upon her death Dr. Alzheimer, now in his Munich laboratory, investigated the patient's brain employing new silver-staining histological techniques to report the distinctive amyloid plaques and neurofibrillary tangles that continue to define AD pathologically. “*A peculiar severe disease process of the cerebral cortex*” was the title of Dr. Alzheimer's case report. The significance of these autopsy findings was not fully appreciated for many years, but the first diagnosis of AD was made. Now over a century later, brain autopsy remains the only way to diagnose definite AD (since brain biopsy is *not* a standard clinical practice).

Why do individuals with AD and similar progressive dementias often go undetected and undiagnosed? Diagnostic difficulties may be due in part to the variability of symptoms, some of which are difficult to identify. Also, dementia may be confused with other conditions including delirium, depression, anxiety, sleep disorders, side-effects of prescription and over-the-counter drug, drugs and alcohol abuse, and post-concussive syndrome. In addition, the onset of dementia is gradual and insidious, and patients may deny—or lack awareness of—their cognitive deficits. Moreover, given the global and regional variability in medical practice and cultural norms, consensus criteria for diagnosis remain controversial. Recent efforts in standardization of definitions and normal (vs. abnormal) values have resulted in greater harmonization of best practice. Controversies remain, and include the answer to questions such as: How much memory decline is acceptable and considered “normal aging”?

The diagnosis of a dementing illness is based on clinical signs and symptoms. After a medical history and physical examination, including a neurologic and psychiatric assessment, procedures employed to diagnose dementia may include neuropsychological testing, laboratory tests (blood and other biologic fluids), brain neuroimaging, and genetic testing. Methods for detecting changes in brain function and physiology are positron emission tomography (PET) and single photon emission computed tomography (SPECT) (1), which have been utilized in some clinical trials along with blood biomarkers. In some cases structural and functional magnetic resonance imaging (MRI), and to a lesser extent, computed tomography (CT) may be used as well. Typically, in the US and Canada, family medicine physicians, but more likely neurologists, geriatric psychiatrists, and geriatricians are trained in diagnosing individuals with dementia. A clinical diagnosis is important in order to determine prognosis, clinical management (including guiding appropriate prescription medications), genetic implications for family member, and clinical trial participation.

New recommendations to improve dementia detection and diagnosis were introduced by a committee of experts at the Alzheimer's Association's International Conference in Chicago, IL, USA, 2018. The steering committee known as *The Consortium for Detecting Cognitive Impairment, Including Dementia* (DetectCID; https://www.detectcid.org/), was formed by the NIH with a goal to establish, test, and validate methods for detecting cognitive impairment in the public, including underrepresented populations. The purpose of this review is to survey *novel* methods and discuss potential challenges that clinicians face with regard to dementia diagnosis at clinical sites. Adoption of any novel methodology will be limited by practice standards, federal, state/provincial, and local government regulations, cost, and third-party coverage.

## Biomarkers

Accumulating data has focused on discovering, evaluating, and validating biomarkers for application in clinical research. The goal in many cases is to provide evidence for earlier diagnostic and prognostic capability. Biomarkers are also employed to confirm and improve on diagnostic accuracy of dementia. In AD, biomarker development and validation has focused primarily on cerebrospinal fluid (CSF) -omics, including proteomics, and PET ligands to detect CNS amyloid beta (Aβ) or tau/tangles—the two pathological hallmarks of AD (2–4). However, the use of CSF and PET biomarkers is limited by their invasiveness and cost, respectively. Other challenges concerning biomarker discovery and validation include collection methods, processing procedures, sample storage, and assay standardization within and across laboratories.

In response to these criticisms and concerns, an international working group (Alzheimer's Precision Medicine Initiative) was formed to review the current state-of-art for blood- based AD biomarkers (2). These would be preferable given that blood tests are more feasible in world-wide settings, are less costly (compared to PET) and less invasive (compared to lumbar puncture for CSF collection). To date, 19 blood-based biomarkers were selected by the working group for additional consideration for AD detection. This working group also outlined a pathway from biomarker identification and development to validation so that academic-industrial partnerships in cooperation with regulatory bodies may co-develop putative blood-based AD biomarkers. Validation of biomarkers should start with assessment in a “black and white” panel study. Samples from patients with a diagnosis of AD would be compared to samples in neurologically healthy controls. This “black and white” study would aim to establish a concordance between the novel biomarker and the standard measure. This would be an attempt to validate the overall accuracy of the biomarker in a known group design. The next step is attempting to replicate the results in a set that more accurately reflects primary care. This second study would implement the technology from the developing laboratory and would involve technology transfer to an existing diagnostic assay that is widely available. The next step is refining the diagnostic algorithm, which would allow a case for potential regulatory approval. Finally, to establish interlaboratory replication, samples would be assayed on the intended equipment for regulatory approval. An optional step is the validation using CSF samples obtained from the same patients in the first study (2). Big data initiatives based on -omics data (Big Data Research and Development Initiative) analyzes large multidimensional blood- based omics data—allowing stratification of populations into well-defined subgroups (sharing commonalties) that may accelerate progress in biomarker development.

There are many challenges related to the validation of blood based biomarkers for dementia. Of 196 candidate blood based biomarkers, only 19 were prioritized for future consideration by the Alzheimer's Precision Medicine Initiative (2). However, none of the 19 blood based biomarkers were deemed to meet the target product profile. Most biomarker candidates were limited by lack of validation in external cohorts. The lack of external validation may lead to selective reporting and inflated predictive accuracy (5). Close cooperation is needed among academia, industry, and regulators to accelerate development of blood based biomarkers for clinical use. Biomarkers are often identified in academia and commercialization is executed by industry. Collaborations between academia and industry would allow for sharing of product testing, access to clinical data, and clinical endpoints (2).

### A/T/N System

Some leading investigators propose that 7 biomarkers may be grouped into 3 categories based on their pathophysiology—the so-called A/T/N system (6) where “A” refers to Aβ/amyloid based markers, “T” to tau/neurofibrillary pathology, and “N” to neurodegenerative or neuronal injury markers. This system uses the three categories and rates each category as either positive or negative. For example, a score could be A+/T+/N-, which would indicate the person is positive for Aβ and tau pathology, but negative for markers of neuronal injury or neurodegeneration.

The 7 biomarkers that are grouped into 3 binary categories include higher Aβ/amyloid deposits measured with PET tracer (7), and low CSF Aβ (8–10). These biomarkers also include tau pathology with greater neurofibrillary tangles in CSF phosphorylated tau and a PET tracer of tau (9, 11). Finally, biomarkers of neurodegeneration or neural injury include total tau in CSF, hypometabolism measured with [18F]-fluorodeoxyglucose ^18^(FDG)-PET, and atrophy on structural MRI in the hippocampus (12).

Biomarkers exist on a continuous scale from normal to abnormal demarcations to have diagnostic categorization of individuals informative for clinical decision making. These demarcations can be arbitrary and many individuals will have biomarkers close to the demarcations, which is true for most diseases and is not unique to AD. The current biomarker measures are not sensitive to low but perhaps clinically significant levels of early pathology (13, 14). The ± binary distinction is a convenient shorthand to increase communication that is easy to use and understand.

## Neuroimaging

A variety of neuroimaging modalities have been developed with the goal of detecting dementia earlier along the AD spectrum and discriminating among the dementia differential diagnosis. The Alzheimer's Disease Neuroimaging Initiative (ADNI) (15) connects researchers across the US and Canada to collect, validate, and use pooled data (and samples), including MRI and PET neuroimaging, genetic data (genome-wide association study or GWAS), cognitive testing, CSF -omics, and blood-based biomarkers. Although the leading neuroimaging methods are PET and MRI, other modalities are employed such as computed tomography (CT) (16, 17)]. Although CT is considered less sensitive than MRI for studies in dementia, CT is particularly useful for detecting bone lesions and new hemorrhage. Other advantages of CT over MRI include lower cost, shorter acquisition time, and no contraindication with claustrophobia or implanted metallic devices such as a pacemaker. SPECT, a nuclear imaging technique integrating CT and radioactive tracers, is also used in dementia diagnosis including, for example, differentiation of FTD from Jakob-Creutzfeldt Disease (JCD) (18). Functional MRI (fMRI) is a non-invasive technique that measures brain activity indirectly via changes in blood oxygenation. Functional MRI is useful in assessing integrity of brain networks in prodromal stages of AD, thus detecting mild cognitive impairment (MCI) (19, 20) and in discriminating LBD from AD (21).

## Transcranial Magnetic Stimulation

Transcranial magnetic stimulation (TMS) is a non-invasive therapeutic approach that uses a changing magnetic field to stimulate underlying nerve cells. TMS is under investigation for the treatment of a variety of neurological disorders, including dementia. Benussi et al. found that paired-pulse TMS distinguishes AD from FTD and healthy controls (HC) (22). In this study (*n* = 175 enrolled and underwent testing), TMS differentiated FTD (*n* = 64) from AD (*n* = 79) with a sensitivity of 91.8% and specificity of 88.6%. The authors propose that the observed difference was based on the activity of different intracortical circuits (i.e., cholinergic, GABAergic, and glutamatergic) in AD vs. FTD patients (both groups with mild disease). In other words, by using different TMS paradigms [short-latency afferent inhibition (SAI), short-interval intracortical inhibition (SICI), and intracortical facilitation (ICF)] one may assess the integrity of cholinergic, GABAergic, and/or glutamatergic cortical circuits. Overall, AD and FTD appeared to differ mainly in SICI-ICF and SAI activity where distinguishing AD and FTD from HC (*n* = 32), resulted in a diagnostic accuracy of >85%.

TMS has also been used to discriminate between atypical Parkinsonian disorders (APD) and AD. In, Benussi et al. (23), APDs such as dementia with Lewy bodies (DLB; *n* = 27), progressive supranuclear palsy (PSP; *n* = 13) and corticobasal syndrome (CBS; *n* = 12) were compared against AD (*n* = 63) and healthy controls (HC; *n* = 39). Similar to the TMS study discussed above, an f intracortical circuit activity using TMS paradigms was examined in these different groups. In this study, an overall diagnostic accuracy of 88.3% was found—with individual diagnostic accuracies as follows; 90.5% for AD, 85.2% for DLB, 76.0% for CBS-PSP, and 94.9% for HCs. Collectively these data suggest that TMS may be useful as a diagnostic tool to discriminate amongst various forms of dementia and other neurodegenerative disorders.

## Electroencephalography

Electroencephalographic (EEG) recordings have also been assessed for diagnosing dementia. EEG records electrical activity of cortical neurons and thus indirectly represents underlying brain function. EEG recording abnormalities are found in subcortical dementias, for instance, in DLB and PDD. Similar to other methods, the goal is to achieve earlier diagnosis with EEG, which is also a non-invasive technique. However, unlike PET or MRI scanning, EEG recordings are comparatively inexpensive and widely available at clinical centers. EEG methods are sometimes divided into two approaches. The first is accomplished in the resting state (awake at rest) in the absence of any stimulus. Since the patient is not required to perform a behavioral task, it is more comfortable and less stressful for patients (24). There are four effects of AD that have been reported in repeated studies in resting state EEG (25). There is a slowing of the power spectrum from high frequency (alpha, beta, gamma) to a low frequency in patients with AD (26). The shift from higher frequency to lower frequency is proportional to the progression of AD. There is a reduction of EEG signal complexity in patients with AD, which is likely caused by neuronal death (27). Decreased in synchronization is observed in patients with AD, which is a result of decreased connectivity between brain areas (28, 29). The cause of desynchronization is not well understood, it may emerge from atrophy of neural networks. There are neuromodulatory deficits in AD patients with their cross frequency interaction (30). For example, beta rhythms modulated at a theta rate is more pronounced in controls than in AD patients.

The second approach to EEG studies is conducted when the subject is performing a pre-defined task (task-oriented). This approach of task-oriented EEG studies is not ideal for most people with AD since patients have an increase of anxiety and anger. Therefore, performance of simple behavioral tasks may result in discomfort and inability to complete the task (31). In a study by Fraga et al. (32), EEG was used to discriminate among elderly healthy controls (HC; *n* = 27), MCI (*n* = 21) and AD (*n* = 15). This study used EEG analysis during an executive function task (a working memory task). Significant differences were found and EEG was suggested to be useful for early MCI diagnosis, for improved AD diagnosis, and for assessing the probability of MCI progression to AD.

The N100-P200 is elicited by presentation of a stimulus in the absence of task demands representing sensory processes as well as attention and peaks around 200 ms (33). While the traditional view was that the N100-P200 was mostly unaffected and therefore not a good biomarker of AD, others have shown significantly longer latencies for the N100-P200 in familial AD (34). This indicates basic sensory and attentional processes may be compromised in AD.

Other forms of EEG have been tested for diagnosing dementia including quantitative electroencephalography (qEEG)—a computer-based method independent of traditional visual and subjective clinician's interpretation and based on statistical pattern recognition. Studies to date show a high diagnostic value of qEEG when evaluating subjects with AD, MCI, and other types of dementia. For example in a 2015 study by Engedal et al. (35), qEEGs distinguished AD patients from control subjects with a sensitivity of 84% and a specificity of 81%. The qEEGs also separated patients with LBD or PDD from AD with a sensitivity of 85% and a specificity of 87%. This study used a statistical pattern recognition method to analyze qEEG with a user-friendly score extracted from multiple qEEG features. The user-friendly features of this statistical pattern recognition would allow for translation into the clinical setting. The statistical pattern recognition method poorly separated patients with AD from those with MCI. However, in a more recent study by Hogh and colleagues (36), qEEG was used as a diagnostic tool in MCI (*n* = 56) and AD subjects (*n* = 32) vs. health controls (HC; *n* = 41) across several sites in Denmark, Norway, and Sweden. Since the diagnostic and prognostic abilities in this study were low, it would not be appropriate for translation into a clinical setting. Overall however, the statistical pattern recognition method used in qEEG was superior to traditional EEG analysis. Also, the qEEG method correlated well with CSF AD biomarkers, suggesting an association with AD pathologies.

## Electrovestibulography

Electrovestibulography (EVestG) is a vestibular-based diagnostic test that measures field potential activity recorded in the external ear canal in response to vestibular stimuli (37). The EVestG test is very similar to electrocochleography, but with the acoustic input replaced by a series of mechanically-driven orthogonal tilts accomplished by having the subject sit in a tilt chair (tilts in 2 dimensions—left/right and forward/backward). Recordings are made when the chair is static and also while moving. To date, EVestG methodology has been applied toward diagnosis and discrimination of PDD (Dastgheib et al. *Med Biol Eng Comp*, in press) vs. other neurological disorders, such as schizophrenia, depression, and Meniere's Disease (38–40). Overall, sensitivities and specificities have been typically above 85%. EVestG is more than 95% accurate in PDD diagnosis in patients that were at different stages of the disease (41). EVestG may provide a quick and non-invasive screening tool for PDD. Given the accuracy of PPD and PDD diagnosis, future research using EVestG should be conducted in other neurodegenerative disorders.

## Clinical Decision Support

Clinical support systems include computerized alerts, clinical guidelines, patient data reports, documentation templates, reference information, artificial intelligence (AI), automated historical comparisons, and diagnostic support tools (42–45). In particular, computer-based clinical decision support systems have evolved as a *high tech* tool for the objective evaluation and comparison of data for diagnostic purposes (45, 46). One example is the PredictND tool (47) that was recently tested in the Amsterdam Dementia Cohort (*n* = 504). In this 10 year study, PredictND was highly accurate in separating several types of dementias from each other (i.e., AD, FTD, DLB, or VaD) and from their respective controls (balanced accuracy 82.3%). In addition to the predicted type of dementia, it also provided a confidence measure for classification. Accuracy was highest for VaD and lowest for DLB. In another recent study (48) across several sites in Europe, PredictND was used to differentiate among groups categorized as subjective cognitive decline [SCD; *n* = 252)], AD (*n* = 138), DLB (*n* = 20), FTD (*n* = 34), and VaD (*n* = 23). In this study, 747 patients completed follow-up visits. Of note, the etiological diagnosis changed in 13% of all cases when using PredictND, but the diagnostic accuracy did not change significantly. However, using the PredictND tool increased clinicians' confidence in their dementia diagnosis, indicating that computer-based support systems may assist with clinical decision making. PredictND uses data from neuropsychological tests, MRI, and CSF tests to classify patients according to the disease state index. Prospective studies have possible limitations. The study design was a tradeoff between retaining clinician's impression of patients and minimizing bias from the first to second session. The time between sessions was longer than intended, which may have affected the result. The follow up time was short, especially the evaluation progression of patients with MCI and SCD. However, this approach draws the clinician to data that are most relevant and removes the need to view tens or hundreds of data points individually.

In addition to the disease state index that PredictND used for diagnosis, others have used data from the ADNI to predict progression from MCI to AD (49). The patients underwent neuropsychological testing, MRI scanning, PET scanning, and CSF analysis. The ADNI analyzed MRI and PET scans in MCI patients using the multivariate technique of independent component analysis (ICA). ICA isolates unique features of biomarkers and potentially reveals patterns underlying the imaging data. ICA was able to predict the progression from MCI to AD (50). Support vector machine is a classification algorithm for pattern classification and predicted the progression of MCI to AD (51). However, even with the diagnostic accuracy mentioned in previous studies, clinical support systems are scarce. There is an absence of clear guidelines from regulatory bodies that impedes acceptance of clinical support systems (52). Developers of clinical support systems and its users should propose guidelines that will standardize clinical support systems. Input from both developers and users may result in more clinicians implementing clinical support systems.

Recently, AI applications have been growing. Deep learning is a type of AI that is sometimes described as simulating human learning approaches. Traditional PET image analysis requires an evaluation by experts trained in nuclear medicine and neuroimaging to make pattern recognition decisions. Therefore, deep learning algorithms theoretically may be used to learn and detect features or patterns in PET scans. In addition, deep learning may potentially help recognize additional patterns that are not as obvious during a human clinical review of scanned images. However, the usefulness of this task remains to be seen. Also, given that traditional PET scan analyses are labor intensive, deep learning algorithms may shorten overall review time. In a recent ^18^FDG-PET study (53), it was hypothesized that a deep learning algorithm could detect patterns not evident on standard human-based clinical image review. PET images were obtained from the Alzheimer's Disease Neuroimaging Initiative (ADNI) database. The InceptionV3 architecture deep learning algorithm was trained on 90% of the ADNI data set and tested on the remaining 10%, as well as the independent test set. The deep learning algorithm achieved a 82% specificity and 100% sensitivity. These findings imply that not only can deep learning algorithms predict the final diagnosis of AD with high accuracy and robustness, but they may also reduce overall cost due to shorter review times completed in part by machines. However, deep learning algorithms so far have been mainly utilized for the diagnosis of AD (54). Several technical challenges must be overcome to apply deep learning methods to other forms of dementia (54), neuroimaging datasets need a certain amount of labeling time to train a machine learning system, and various types of noise in the images reduces algorithm accuracy, to name a few. Some experts also state the best most AI systems do is reflect the past history's context for the current sample. Finally, once deep learning algorithms are optimized (85–95%) to match specific types of human thinking, there may be no *wiggle* room left for “original” thought. These and other prognostic tools represent valuable support for clinicians. However, it is important to evaluate and compare performance in a standardized manner (55).

## Olfaction and Taste

Humans are capable of growing new nerve cells throughout life in a process called neurogenesis—suggesting a novel treatment strategy for dementia. To date, the two human brain regions that are sites of adult neurogenesis are a subfield of the hippocampus and the olfactory bulb (56). In fact, olfactory disorders may predict pre-dementia and dementia (57). Given this, it was hypothesized that olfaction and neurogenesis may be impaired in those with dementia, and that olfactory disorders may predict the conversion from MCI to AD dementia (57). Presently, no gold standard olfactory test is available for diagnosing or monitoring AD in clinical practice, but efforts have been made for predicting AD and for discriminating dementia diagnoses. For example, Williams et al. (58), found that that olfactory impairment was more pronounced in patients with mild DLB than in those with mild AD. Interestingly, in another study (59) patients with AD may demonstrate an asymmetrical decrement of odor detection sensitivity (left worse than right). In this study, the left-right nostril odor detection test functioned as an inexpensive, sensitive and specific test for probable AD.

The human sense of smell aligns with taste perception, and even vision to some degree (60). More recently (61, 62), taste cognition and taste detection were tested in subjects with suspected dementia. In one such study (62), the hypothesis was tested that the insula is associated with taste cognition in patients with AD (*n* = 30) and VaD (*n* = 20) vs. healthy controls (*n* = 15). Overall, it was concluded that glucose metabolism in the right insula was lower in the low taste cognition cohort and VaD patients with insular lesions showed impaired Taste Cognition Test results. Other recent studies (61) suggest that a failure of CNS taste processing occurs in patients with AD.

## Vision

Vision is impaired in dementia—with a variety of demonstrable impairment including contrast sensitivity (63). More recent studies (64, 65) examined whether a retinal examination may predict AD earlier and reveal disease progression (66, 67). Mahajan et al. (64), found ocular changes in AD besides decreased contrast sensitivity and included decreased vision, abnormal pupillary reaction, visual field changes, loss of retinal ganglion cells (and retinal nerve fiber layer), peripapillary atrophy, increased cup– disc ratio, retinal thinning, tortuosity of blood vessels, and the deposition of Aβ in the retina.

Examining color vision is also a potentially useful tool for discriminating different types of dementia. For instance, color vision discriminates AD from DLB (68). In this case, it was concluded that color vision deficits in patients with DLB showed a prevalence similar to the defining core features of DLB (~80%) and may be supportive of a diagnosis of DLB compared to AD. Other studies (69) found color vision differences when comparing AD to VaD. In this study, the sensitivity/specificity analysis was 80.6% and 87.5% for discriminating AD vs. VaD.

Beta-amyloid deposits are found in the retina of patients with AD and are associated with a narrowed lumina and occlusion (70–73). Retinal photography was able to distinguish patients with AD and non-AD with 100% sensitivity and 84% specificity (74). The amyloid levels detected in the retina were correlated with amyloid levels in the brain via PET scan. An increase of 3.5% in retinal amyloid during a 3.5-months period suggests that retinal imaging could be used for monitoring the response to treatment (64). The retinal amyloid test is a screening tool that could complement currently used tests and potentially be used as part of regular eye exams.

## Saliva

Using saliva samples to diagnose AD has several advantages such as the non-invasive ease of acquisition and low cost. Chertkow et al. (75) used saliva and immunoblot analysis to quantify the phosphorylated tau (p-tau)/total tau (t-tau) ratio at different phosphorylation sites. Hyperphosphorylated tau (indicated by p-tau) is a pathological marker for AD. In this study, samples were obtained from AD, MCI, and FTD patients. With one phosphorylation site, Ser-396, the p-tau/t-tau ratio was significantly increased in patients with AD compared with elderly control subjects. However, the sensitivity and specificity were not sufficiently robust to serve as a standard clinical biomarker. In fact, about one third of the AD group failed to show elevations of salivary tau. Another study with saliva (76) measured salivary acetylcholinesterase (AChE) activity in AD—an enzyme deficient in AD patients. The study examined in 15 AD patients who were taking memantine vs. 15 healthy subjects. AChE activity in saliva in the AD group was indeed lower compared to the control group, but there was no significant difference between groups.

## Speech

Speech impairment is well-known in AD (77) and other dementias and impairment in verbal communication depends on AD stage (78). Progression of speech impairments vary by individual, but three stages are identified (78). In the first, subjects demonstrate word-finding difficulties. In the intermediate stage, vocabulary and language become weaker. In the advanced stage, subjects provide only limited answers consisting of a few words. Nasrolahzadeh et al. (78), examined speech in AD subjects with the goal of utilizing spontaneous speech for earlier detection. This study focused on analyzing and comparing the quadratic phase coupling of spontaneous speech signals from healthy controls (*n* = 30) vs. AD subjects (*n* = 60) using bi-spectrum and bi-coherence methods. Signal processing methods of this type are statistical methods utilizing non-linear interactions of a continuous spectrum of propagating waves in one dimension. All participants were asked to tell “*graceful personal stories, express their feelings, and converse in a friendly way*.” The results showed that the spontaneous speech signal of those with AD was significantly reduced compared to healthy controls.

In another study (79), speech samples were compared in probable AD subjects (*n* = 225) vs. probable DLB (*n* = 67) subjects. In particular, speech samples were evaluated using the Cognitive Status Examination [COGNISTAT; formerly the Neurobehavioral Cognitive Status Examination (NCSE)], in which exam takers discuss what is happening between two people in a presented picture; however, other domains in addition to language may be tested, such as constructional ability, memory, calculation skills, and executive skills. During this test, subjects were scored (in a team effort by several psychologists) based on whether the subjects described or did not describe the relationship between two people during the speech sample. For instance, an example of the description group was as follows; “*This is a picture of fishing. Someone is calling over. The person fishing does not notice a fish caught on the hook because he dozed off*.” In the no-description group, a typical answer may be “*A person is fishing. A person is performing acrobatics on the bridge*.” In addition, study participants were tested with the Mini-Mental State Examination (MMSE). The results suggest that patients with more severe overall cognitive dysfunction and also male patients are less likely to describe the relationship between two people. Difficulties with picture naming tasks are one of the most frequently reported speech impairments in people with AD (80).

Verbal fluency tests are one of the most widely used measures of speech function in patients with dementia (81). These tasks assess the person's ability to retrieve and produce words relevant for the specific task. Letter fluency records the generation of as many words as possible beginning with a given letter, for example words that begin with the letter S. Category fluency involves the generation of as many words as possible that fall into a specific category, for example tools. Letter and category fluency place demand on executive functioning since patients must engage in verbal retrieval and recall and inhibit incorrect responses. A meta-analysis of 153 studies with 15,990 AD patients found that AD patients had impaired letter fluency (82). Category fluency declines with the progression of AD (83).

Naming difficulty is another well-documented symptom of AD and it typically occurs early in disease onset (81). The Boston Naming Test (BNT) is a widely used test that comprises 60-items ranging from frequent to infrequent items. The patient is presented with an item and allowed approximately 20 s to verbally identify the item. However, some patients with dementia find the 60-item version difficult to complete due to their limited attention. Therefore, the BNT developed two 30-item versions that significantly correlated to each version and the 60-item version. Differences have been found between patients with MCI and controls (84), and between AD and non-AD individuals (85).

A speech language pathologist can modestly improve communication for people with moderate to severe dementia (86). Speech language therapy may offer some protection against further speech decline. However, future studies should examine this question by measuring speech over a long period of time. Also, caregivers can be trained on the methods used by a speech language pathologist, which would lessen the time the patient must to be in the clinical setting.

## Neuropsychologic Testing

Detailed comparator studies as well as comprehensive reviews addressing tests used for assessing cognitive status in AD and other dementias are published (87–97). Neuropsychologic tests may be organized by cognitive, functional, or behavioral domains (or their combinations) including activities of daily living (ADLQ), short mental status tests (MMSE, MoCA), brief dementia batteries (RBANS), behavioral symptoms (NPI-Q), clinical ratings (CDR), mood (Beck Depression Inventory II), IQ (Wechsler), executive function (Stroop test), visuoperceptual (drawing a clock), language or calculation (BDAE), and episodic memory (paragraph recall, word-list learning, Rey-Osterreith Complex Figure). When administered as a battery, neuropsychological assessments quantify cognitive impairments and rates of progression. The CANTAB is a touchscreen computer automated neuropsychological test battery, which measures learning and memory. Patients with AD are impaired on the CANTAB test battery as compared to controls (98). Virtual reality is used to measure spatial navigation, which is impaired in people with dementia (99). The CANTAB and virtual reality could be integrated into family practice such that any medical professional could administer the task.

## Challenges for Implementation at Clinical Research Sites

The goals of biomarker inclusion in newer diagnostic criteria include making a more accurate diagnosis of dementia. However, multiple barriers to implementation of innovative diagnostic methods and biomarkers limit their clinical application. These barriers include patient access to medical care, feasibility, cost, and third-party coverage.

Many individuals with dementia are *never* diagnosed by clinicians. Diagnostic nihilism stems in part from a widely-held perception that currently available drug treatments for AD are inadequate—that they have minimal, if any, benefits, and that risk-benefit and cost-benefit analyses are negative. This current situation with dementia mimics a long-ago time when clinicians chose not to tell patients if they had terminal cancer. Clinicians should make an accurate dementia diagnosis, or refer to a specialty center as needed. If possible, biomarkers should be added to support a dementia diagnosis—as third-party coverage permits. An accurate diagnosis will determine prognosis, guide clinical care and management, enable a discussion of genetic risk with family members, and raise the possibility of clinical trial participation. Newer diagnostic technologies may be available if an individual screens or enrolls in a clinical study (for example, amyloid and tau PET scans, CSF proteomic analysis of Aβ and tau, and ApoE genetic testing).

The development of newer biomarkers (for example, amyloid PET) has uncovered a 10–20 years prodrome of MCI and AD. This population—cognitively normal but at higher risk for AD—is increasingly targeted for clinical research including prevention trials. The creation of databases composed of at-risk volunteers will aid recruitment for clinical studies. Recent efforts are also building trial-ready cohorts of well-characterized individuals in order to improve clinical trial efficiency and lower the high rates of screen-failure.

A significant challenge to implementation is the validation for use in a family practice setting. Clinical support systems need the guidelines of regulatory bodies and communication between the developers and clinicians to implement systems. There are many steps involved to validate blood based biomarkers including establishing a concordance between the novel biomarker and the standard measure, replication in an external laboratory, refinement, transfer to a commercial platform, and validation in an independent cohort. A similar process would be implemented to validate saliva based biomarkers. The computer based neuropsychological testing with an iPad touchscreen or virtual reality could be administered by any medical professional in the family practice setting.

## Conclusion

In addition to a traditional medical history and neurologic examination, new technologies may assist in the diagnosis of dementia or impending dementia due to neurodegenerative disorders. Newer iterations of diagnostic criteria are incorporating validated diagnostic biomarkers, when available, as supportive evidence of a particular dementia diagnosis. Controversies remain, however, regarding the optimal biomarker, or combination of biomarkers, to include in these criteria. A lack of consensus of expert opinion, however, is not the only limitation to their clinical application. Operational issues including availability, feasibility, cost, and third-party coverage will limit their incorporation into clinical practice. Novel diagnostic biomarkers, particularly if relatively inexpensive and non-invasive, have the potential to markedly improve current practice, with added value in screening, prognosis, accurate diagnosis, and evaluation of novel treatments now under development for dementias including dementia due to AD.

## Author Contributions

RT added a perspective from his memory clinic that he directs. TS added content relevant to CROs. BA who holds 2 dementia research chairs, was responsible for much of the research. DD revised a portion of the manuscript. All authors contributed to the writing of the manuscript.

## Conflict of Interest

RT: research support to Georgetown University from Lillly, Biogen, Roche, Genentech, Novartis, Janssen, and Eisai. TS: owner and CEO of ActivMed Practices and Research, Inc.; owner and CEO of ALLCUTIS Research, Inc.; owner and President of ActivMatters of the Brain, Inc. BA: medical advisor for Clinical Research IO. Medical advisor for Mitochondrial Transfusion. The remaining author declares that the research was conducted in the absence of any commercial or financial relationships that could be construed as a potential conflict of interest.
